# CT-based assessment of sarcopenia for differentiating wild-type from mutant-type gastrointestinal stromal tumor

**DOI:** 10.1038/s41598-022-27213-8

**Published:** 2023-02-24

**Authors:** Xiaoping Yi, Gaofeng Zhou, Yan Fu, Jinchun Wu, Changyong Chen, Hongyan Zai, Qiongzhi He, Peipei Pang, Haiyan Zhou, Guanghui Gong, Tianxiang Lei, Fengbo Tan, Heli Liu, Bin Li, Bihong T. Chen

**Affiliations:** 1 Department of Radiology, Xiangya Hospital, Central South University, Changsha, 410008 Hunan People’s Republic of China; 2 National Clinical Research Center for Geriatric Disorders (Xiangya Hospital), Central South University, Changsha, 410008 People’s Republic of China; 3 Hunan Key Laboratory of Skin Cancer and Psoriasis, Xiangya Hospital, Changsha, 410008 Hunan People’s Republic of China; 4 Hunan Engineering Research Center of Skin Health and Disease, Xiangya Hospital, Changsha, 410008 Hunan People’s Republic of China; 5 Department of Oncology, Xiangya Hospital, Central South University, No. 87 Xiangya Road, Changsha, 410008 Hunan People’s Republic of China; 6 Geneplus-Beijing Institute, Beijing, People’s Republic of China; 7 GE Healthcare, Hangzhou, 310000 People’s Republic of China; 8 Department of Pathology, Xiangya Hospital, Central South University, Changsha, 410008 Hunan People’s Republic of China; 9 Department of General Surgery, Xiangya Hospital, Central South University, No. 87 Xiangya Road, Changsha, 410008 Hunan People’s Republic of China; 10 Department of Diagnostic Radiology, City of Hope National Medical Center, Duarte, CA 91010 USA

**Keywords:** Cancer, Genetics, Biomarkers, Oncology

## Abstract

Non-invasive prediction for KIT/PDGFRA status in GIST is a challenging problem. This study aims to evaluate whether CT based sarcopenia could differentiate KIT/PDGFRA wild-type gastrointestinal stromal tumor (wt-GIST) from the mutant-type GIST (mu-GIST), and to evaluate genetic features of GIST. A total of 174 patients with GIST (wt-GIST = 52) were retrospectively identified between January 2011 to October 2019. A sarcopenia nomogram was constructed by multivariate logistic regression. The performance of the nomogram was evaluated by discrimination, calibration curve, and decision curve. Genomic data was obtained from our own specimens and also from the open databases cBioPortal. Data was analyzed by R version 3.6.1 and clusterProfiler (http://cbioportal.org/msk-impact). There were significantly higher incidence (75.0% vs. 48.4%) and more severe sarcopenia in patients with wt-GIST than in patients with mu-GIST. Multivariate logistic regression analysis showed that sarcopenia score (fitted based on age, gender and skeletal muscle index), and muscle fat index were independent predictors for higher risk of wt-GIST (*P* < 0.05 for both the training and validation cohorts). Our sarcopenia nomogram achieved a promising efficiency with an AUC of 0.879 for the training cohort, and 0.9099 for the validation cohort with a satisfying consistency in the calibration curve. Favorable clinical usefulness was observed using decision curve analysis. The additional gene sequencing analysis based on both our data and the external data demonstrated aberrant signal pathways being closely associated with sarcopenia in the wt-GIST. Our study supported the use of CT-based assessment of sarcopenia in differentiating the wt-GIST from the mu-GIST preoperatively.

## Introduction

Gastrointestinal stromal tumor (GIST) is a common interstitial tumor occurring in gastrointestinal tract^1,2^. Most GISTs harbor KIT/PDGFRA mutant gene named KIT/PDGFRA mutant GIST (mu-GIST), and the remaining cases (about 10–15%) are KIT/PDGFRA wild-type GISTs (wt-GIST) which do not have mutant KIT/PDGFRA genes^2^. It is important to differentiate these two subtypes of GIST for selecting effective treatment^3^. For example, imatinib is an active multikinase inhibitor that mainly targets C-kit tyrosine-kinase receptors. Although imatinib has become the standard first-line treatment worldwide for patients with the mu-GIST, it almost ineffective for patients with the wt-GIST. Clinically, neo-adjuvant therapy with imatinib was strongly recommended for mu-GIST patients prior to surgery with the purpose of sparing important organs which may be locally invaded by tumor^4,5^. Currently, the only method for preoperative molecule identification of the GIST subtypes is through aspiration biopsy, which is invasive and may have potential risk of tumor rupture, perforation of gastrointestinal tract or needle track implantation^6^. There is an unmet need for developing a robust non-invasive tool for differentiating wt-GIST from mu-GIST before treatment.

Abdominal computed tomography (CT) is routinely used for clinic management of GIST. Sarcopenia (loss of lean muscle mass) can be evaluated on the routine abdominal CT images obtained for clinical care^7–9^. A recent study has identified the presence of sarcopenia in GIST patients and highlighted the predictive value of sarcopenia on treatment toxicity^10^. However, there is a paucity of literature regarding clinical value of pre-treatment sarcopenia for patients with GIST regarding their response to treatment and prognosis. Converging evidences have indicated that sarcopenia may possibly underline the pathogenesis of various cancers, because signal pathways involved in sarcopenia are usually anomalous in cancer^8,11^. We hypothesized that different molecular pathways for wt-GIST and mu-GIST may lead to significant differences in muscle mass of patients with different subtypes of GIST.

Here, we retrospectively analyzed the lean muscle mass on abdominal CT images of 174 patients with GIST and evaluated whether CT assessment of sarcopenia could differentiate the wt-GIST from the mu-GIST. In addition, we also performed genetic analysis on both our own data and an external dataset.

## Materials and methods

### Patients

This single-center retrospective study including all experimental protocols, was approved by the Institutional Review Board of Xiangya hospital (IRB#: 202009685). The written informed consents were waived by the Institutional Review Board of Xiangya hospital due to the retrospective nature of this study. All methods were carried out in accordance with relevant guidelines and regulations.

**Figure 1:**
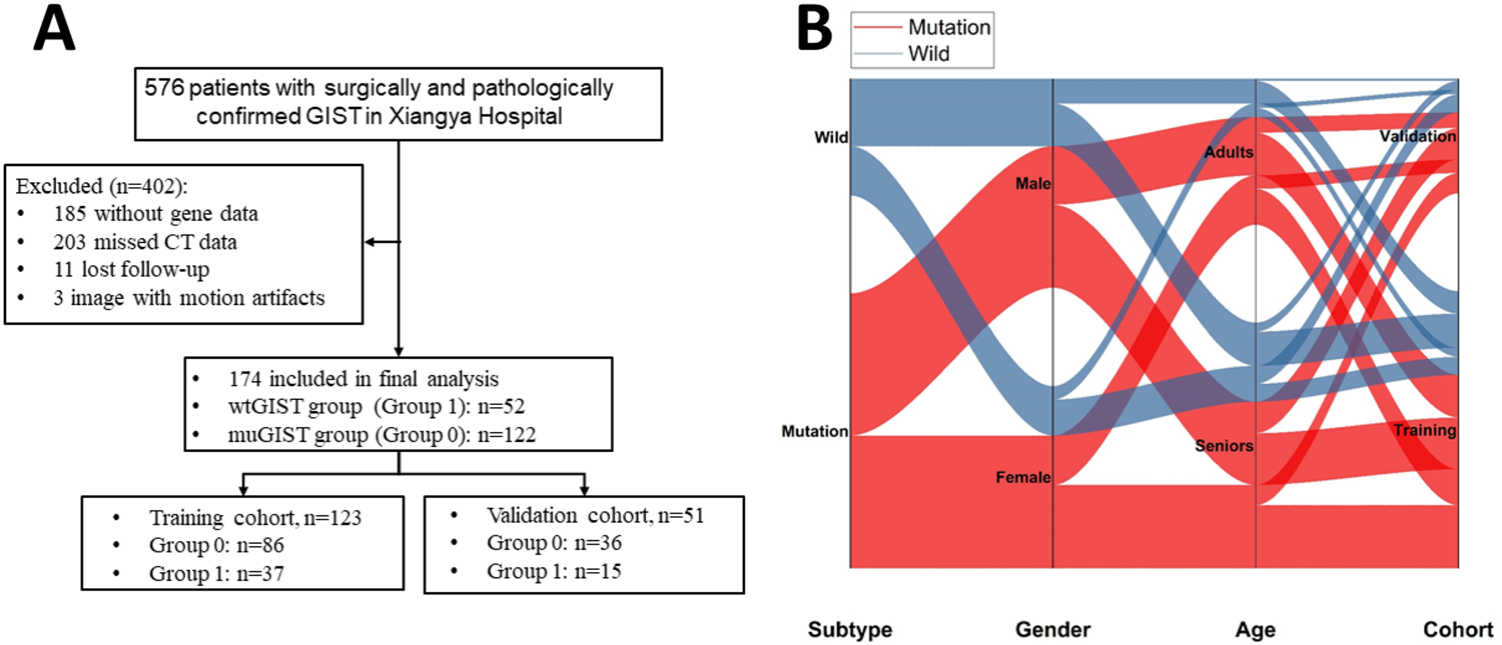
(**A**) Flow-chart demonstrating the patient recruiting process and the exclusion criteria. (**B**) Sankey diagram showing the scmap cluster projection of the GIST subtypes, gender, and age.

Patients with pathologically proved GIST and with genotyping information were retrospectively identified and their data was collected after searching our institutional medical database from January 2011 to October 2019. This cohort was divided into two groups: the wt-GIST group and the mu-GIST group. Details of the patient recruiting process and exclusion criteria were presented in Fig. 1. Next, the patients were randomly selected into a training cohort and a validation cohort at a ratio of 7:3 for future analysis.

### Re-analysis of pathology specimen

For each patient, all pathological slides were re-analyzed by two pathologists specialized in GIST (HYZ and GHG with 17 and 6 years of experience in solid tumor, respectively). Consensus was reached by discussion if differences in opinions occurred. KIT and PDGFRA gene were amplified and sequenced by Sanger sequencing set kit according to the manufacturer’s instructions using primers designed for the c-KIT exon 9, 11, 13, 14, 17, 18 and PDGFRA exon 12, 18.

### Body composition evaluation

**Figure 2:**
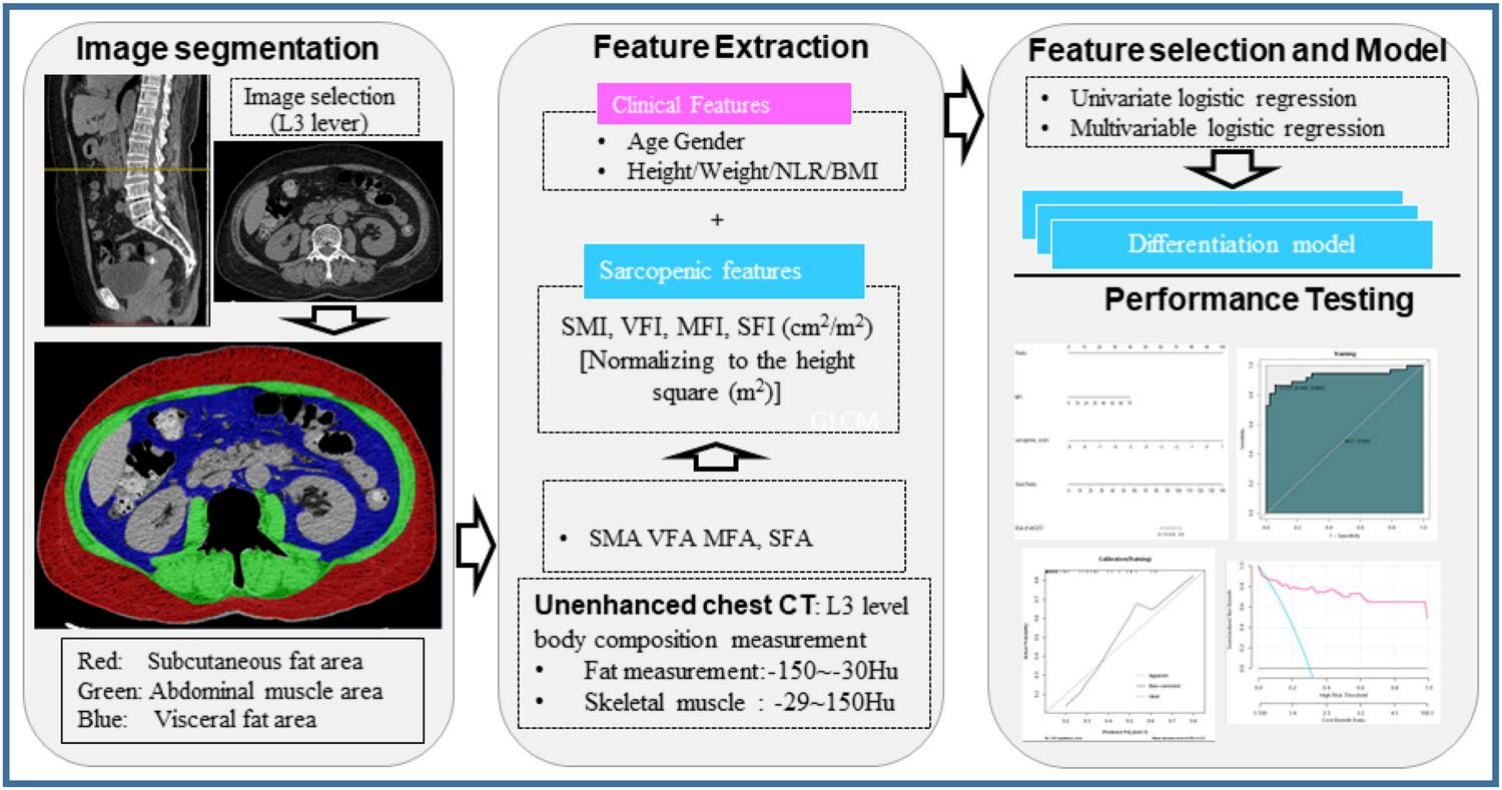
Workflow for body composition measurement on the abdominal CT images. *VFA* visceral fat area, *MFA* fat area in muscle, *SFA* subcutaneous fat area, *SMA* skeletal muscle area, *SMI* skeletal muscle index, *VFI* Visceral fat index, *MFI* muscle fat index, *SFI* subcutaneous fat index.

Initial pre-treatment abdominal CT images were retrieved from Picture Archiving and Communication Systems (PACS) for analysis. Two axial unenhanced images were selected at the level of the third lumbar vertebra body (for measurement of skeletal muscle) and at the navel level (for measurement of visceral fat) respectively. Total body fat area, visceral fat area (VFA), fat area in muscle (MFA), subcutaneous fat area (SFA), and skeletal muscle area (SMA) were measured manually on the selected axial image by using a medical image analysis software (SliceOmatic software; Tomovision, Quebec, Canada). Bone structures, and the central spinal canal were excluded manually in the selected axial images for accurate measurement of specific areas on a workstation (Advantage Windows workstation 4.6, GE Healthcare, Milwaukee, Wisconsin, USA). According to a prior study^10^, corresponding fat area (between − 50 and − 150 Hounsfield units, Hu) and the area of the abdominal wall and back muscles (psoas, paraspinal, transversus abdominis, rectus abdominis, internal oblique muscles, and external oblique muscles) (between − 29 and 150 Hu) were measured by calculating the area of pixels with corresponding attenuation in demarcated areas, respectively. L3 skeletal muscle index (SMI) was calculated as the area of total L3 skeletal muscle (cm^2^) divided by height square (m^2^). Visceral or subcutaneous fat index (VFI, MFI or SFI) was calculated as the area of visceral, intramuscular or subcutaneous fat (cm^2^) at the navel level divided by the square (m^2^) of height. The details for body composition measurement was presented in Fig. 2.

### Treatment, follow-up and outcome parameters

All patients underwent surgery, and post-surgical follow-up was obtained for each patient. The primary endpoints of the study were disease free survival (DFS). DFS was defined as the time from surgery to recurrence of GIST or metastasis for expired patients or the last follow-up for surviving patients.

### Genomic data analyses

Genomic DNA data for 28 patients in our cohort including 15 wt-GIST samples (sanger sequencing negative for c-KIT exon 9, 11, 13, 14, 17, 18 and PDGFRA exon 12, 18) and 13 KIT mu-GIST samples (sanger sequencing positive for c-KIT exon 11) were obtained from our archived formalin-fixed paraffin-embedded (FFPE) tissues. Ten peripheral blood lymphocyte (PBL) samples from the 15 wt-GIST patients were collected as germline controls. The comprehensive genetic alterations of these 28 GIST samples were analyzed using targeted deep sequencing of 1021 cancer related genes region. The Next Generation Sequencing was executed according to the manufacturer’s instructions (Illumina, San Diego, CA, USA). The somatic mutations of the wt-GISTs were investigated for genomics landscape description with the ComplexHeatmap R package.

In addition, mutational landscapes and corresponding clinical information were downloaded from cBioPortal as external data (http://cbioportal.org/msk-impact). Data was analyzed by R version 3.6.1 and KEGG enrichment analysis was performed by clusterProfiler. The pathway with p-value less than 0.05 were kept for the following comparison analysis.

### Statistical analysis

Statistical analysis was implemented with SPSS 22.0, SAS9.4, and R software (http://www.Rproject.org). Multivariate binary logistic regression, nomograms, calibration plots, decision curve and correlation matrix plots were done with the “Caret”, “PROC”, “rmda”, “corrplot”, “forestplot”, “rms” and “corrplot” package. Decision curve was performed with the “rmda” package. And “survival” package was used for the survival analysis. KEGG analysis^12^ was performed by R version 3.6.1 through application of clusterProfiler. The statistical significance levels were all two-sided with statistical significance set at 0.05.

### Ethical approval

Institutional Review Board approval was obtained (IRB#: 202009685).

### Contribution to the field statement

We assessed body composition features and sarcopenia on the pretreatment abdominal CT images in patients with Gastrointestinal stromal tumor (GIST). Our prediction model combining sarcopenia parameters and clinical variables showed robust performance, indicating the potential for using such a non-invasive method for predicting KIT/PDGFRA status.

## Results

### Patients’ clinicopathological information

In total, 174 consecutive patients with GIST were included into our study. The clinicopathological characteristics of the patients in the wt-GIST group and mu-GIST group were summarized in Table 1. The medium Neutrophil lymphocyte ratio (NLR) value of the patients with wt-GIST was slightly higher than that of the patients with mu-GIST. There were no significant differences in the clinical characteristics between the wt-GIST group and the mu-GIST group, either in the training cohort or in the validation cohort.

**Table 1:** Clinical and body composition data.

	wt-GIST (n = 52)	mu-GIST (n = 122)	*P*
Age (medium, min–max) (year)	55.0 (28.0–79.0)	54.0 (22.0–84.0)	0.782
Gender (male, %)	30 (57.7)	63 (51.6)	0.464
NLR (medium, min–max)	2.98 (1.15–32.67)	2.55 (0.87–27.00)	0.045*
BMI (mean ± SD)	21.90 ± 2.92	22.45 ± 3.04	0.272
VFA (medium, min–max)	155.75 (10.96–309.91)	81.87 (5.37–247.39)	< 0.001**
VFI (medium, min–max)	57.38 (3.89–123.76)	31.55 (2.02–89.78)	< 0.001**
MFI (medium, min–max)	49.57 (0.17–69.33)	2.74 (0.52–10.19)	< 0.001**
SFI (medium, min–max)	114.94 (8.70–209.14)	46.42 (1.36–129.03)	< 0.001**
VFI: SFI ratio (medium, min–max)	0.50 (0.16–1.44)	0.68 (0.17–1.85)	0.008**
SMI	41.02 (28.53–57.51)	43.28 (25.32–113.26)	0.022*
Obesity (n, %)	12, 23.1	37, 30.3	0.33
Sarcopenia (n, %)	39, 75.0	59, 48.4	0.001
Visceral obesity (n, %)	40, 76.9	45, 36.9	< 0.001**

*NLR* Neutrophil to lymphocyte ratio, *SD* standard deviation, *BMI* body mass index, *VFA* Visceral fat area, *VFI* Visceral fat index, *MFI* Muscle fat index, *SFI* Subcutaneous fat index, *SMI* Skeleton muscle index, *Min* minimum, *Max* maximum. *P < 0.05; **P < 0.01.

### Body composition status for GIST subtypes

The VFA, VFI, MFI and SFI of patients in the wt-GIST group were all significantly larger than those of patients in the mu-GIST Group. The SMI and VFI/SFI Ratio of the wt-GIST Group were significantly lower than those in the mu-GIST Group (Table 1).

Taking into considerations of the influence of age and gender on skeletal muscle index (SMI), we developed a new index termed sarcopenia score based on the three features through multivariate logistic regression. Not surprisingly, the sarcopenia score for the wt-GIST group (− 0.597, [− 8.940 to 1.620]) was significantly higher than that of the mu-GIST group (− 1.060, [− 2.470 to 0.900]) (*P* < 0.001).

There was no significant difference on obesity (calculated based on BMI) between the wt-GIST group and the mu-GIST Group (23.1% vs 30.3%) (*P* = 0.33). The incidence of sarcopenia and visceral obesity in the wt-GIST group was much higher than that of the mu-GIST group (75.0% vs 48.4%, 76.9% vs 36.9%, respectively) (*P* < 0.001). Interestingly, the inconsistent diagnosis of adiposity, including obesity diagnosed based on BMI and visceral obesity based on VFA, occurred in 32.18% (56/174) of patients with GIST. BMI was normal in forty-six GIST patients with visceral obesity, while ten obese patients did not actually have visceral obesity.

### Identification of risk factors associated with wt-GIST

Single factor logistic regression analysis showed that the patients with wt-GIST were associated with NLR, VFA, MFA, SFA, VFA/SFA ratio, SMI, VFI, MFI, SFI, sarcopenia score, sarcopenia and visceral obesity. Further multivariate logistic regression analysis showed that, only sarcopenia score (*P* = 0.000298, OR 10.216 [95% CI 3.207–41.151]) and MFI (*P* = 0.005, OR 1.141 [95% CI 1.079–1.380]) were identified as independent risk factors for wt-GIST.

### Development and validation of individualized prediction model

**Figure 3:**
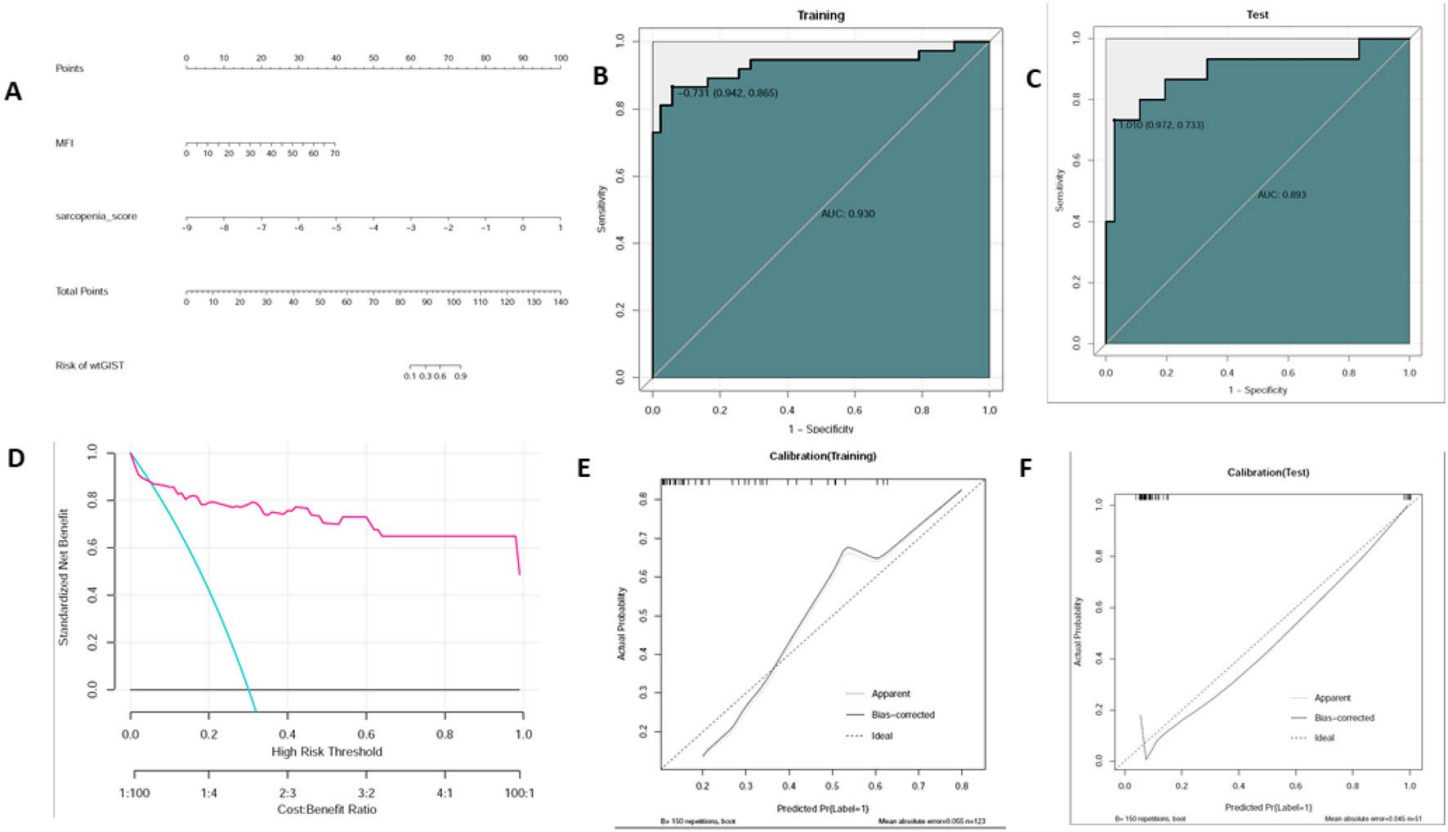
(**A**) Sarcopenic nomogram. The nomogram was developed in the primary cohort, with the sarcopenia score and MFI incorporated. The classification efficiency of the nomogram was shown by ROC curves for training (**B**) and validation (**C**) cohorts respectively. Calibration curve of the nomogram in the primary cohort (**E**) and the validation cohort (**F**).

A parsimonious model separating the patients with wt-GIST from the patients with mu-GIST was developed by incorporating the two independent predictors (sarcopenia score and MFI), and a nomogram was obtained (Fig. 3A). The ROC test provided an AUC of 0.879 (95% CI 0.816–0.943) with a sensitivity of 0.8780 (95% CI 0.7561–0.9756) and a specificity of 0.9167 (95% CI 0.8611–0.9722) in the training cohort (Fig. 3B), and a AUC of 0.9099 (95% CI 0.8324–0.9873) with a sensitivity of 0. 9412 (95% CI 0.7647–1.0000) and a specificity of 0.8871 (95% CI 0.7742–0.9839) in the validation cohort (Fig. 3C).

The calibration curve of the nomogram for the probability of wt-GIST showed good agreement between prediction and observation in both the training and validation cohorts (Fig. 3E,F). The Hosmer–Lemeshow test yielded a non-significant statistic in both the training (*P* = 0.819) and the validation cohort (*P* = 0.751), which suggested no departure from perfect fit.

The decision curve analysis for the nomogram was presented in Fig. 3D. The decision curve showed that if the threshold probability of a patient or doctor was 10%, using the nomogram to predict wt-GIST added more benefit than either the diagnose-all-patients scheme or the diagnose-none scheme.

### Survival analysis

Seven (13.5%, 7/52) patients were lost at the median follow-up of 31.0 months (range 1.0–73.0 months). During the follow-up period, only 3 (6.67%, 3/45) and 4 (8.89%, 4/45) deaths were observed, respectively. The relevant prognostic analysis was not completed because the majority of patients did not have an endpoint event. However, it should be noted that all endpoints (recurrence or death) occurred in patients with sarcopenia and visceral obesity. The incidence of recurrence was higher in patients who were sarcopenic compared with those who were not (11.4% vs. 0%), although the difference was not statistically significant due to the relatively short follow-up period (*P* = 0.561). Furthermore, two patients with visceral obesity could not be diagnosed as obesity based on their BMI values. The impact of sarcopenia and visceral obesity rather than obesity on the survival of wt patients with -GIST (DFS, OS) were observed in the log-rank test, although there was no statistical significance (Supplementary Fig. 1).

### Abnormal gene pathways in GIST subtypes

**Figure 4:**
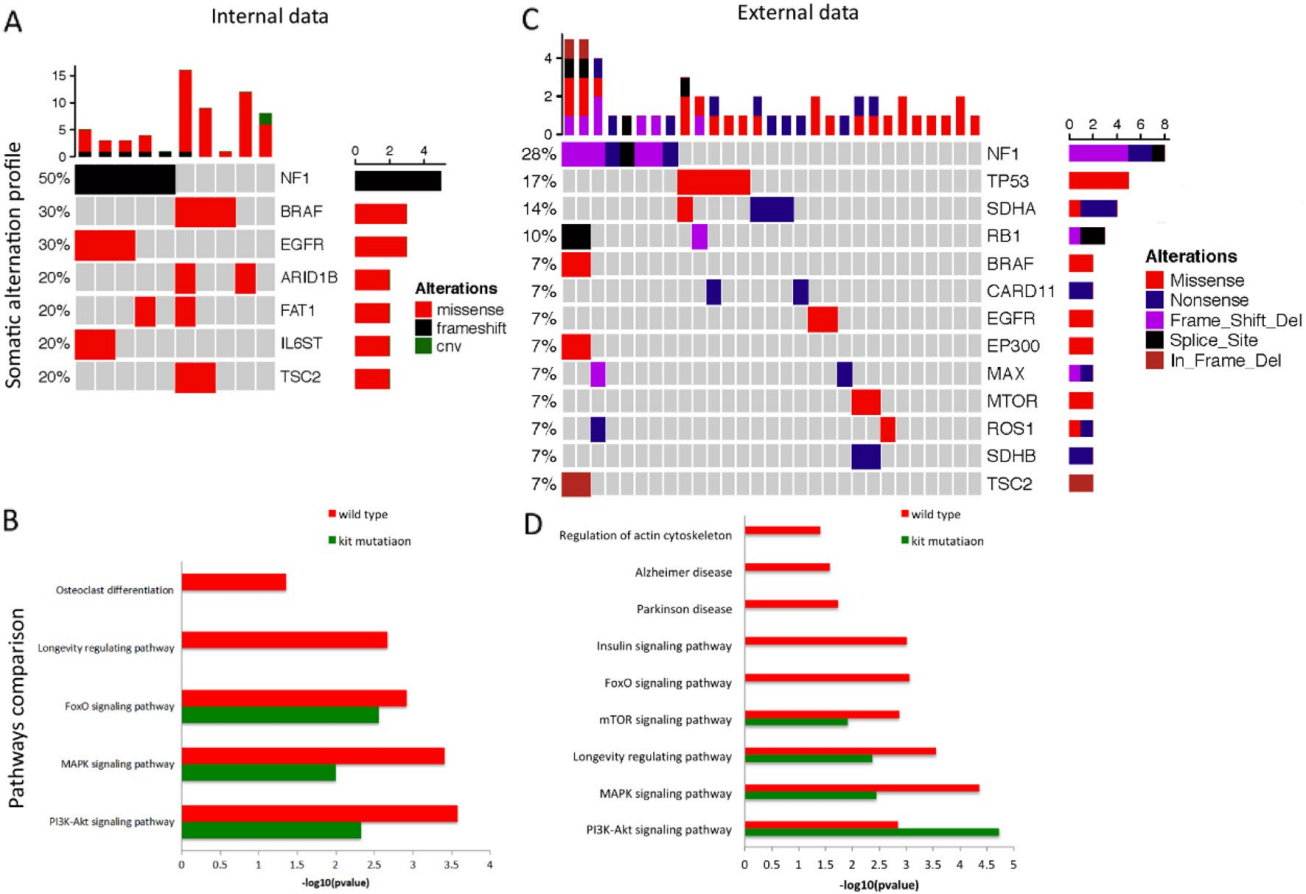
Hot map and KEGG analysis in wt-GISTs in our center as internal data and in Memorial Sloan Kettering Cancer Center (MSKCC) database as external data. (**A**,**C**) Hot map of wt-GISTs (**A**: internal data, **C**: external data). Patients were arranged along the x-axis. Mutation per Mb region was shown in the upper panel. Genes with somatic mutations (The gene mutation >  = 2 patients were included) were shown in the middle panel. Mutation frequencies of each gene were shown on the left. The type of alternations was shown in the right. (**B**,**D**) Enriched KEGG pathway comparison between wt-GISTs and mu-GIST (**B**: internal data, **D**: external data). Y-axis representing the enriched KEGG pathway; X-axis representing the value of − log10 (p-value).

As shown in Fig. 4A, in our center, the frequently mutated genes in the wt-GIST (occurred in at least 2 cases) were neurofibromatosis type 1(NF1) (5/10), B1 v-raf murine sarcoma viral oncogene homolog B1 (BRAF) (3/10), Epidermal growth factor receptor (EGFR) (3/10), AT-Rich Interaction Domain 1B(ARID1B) (2/10), FAT Atypical Cadherin 1(FAT1) (2/10), interleukin 6 signal transducer(IL6ST) (2/10) and tuberous sclerosis2(TSC2) (2/10).

Kyoto Encyclopedia of Genes and Genomes (KEGG) analysis indicated that the pathway enriched in osteoclast differentiation and longevity regulating pathway was noted in the wt-GISTs. When compared to the mu-GISTs, higher forkhead box O (FoxO) signaling pathway and its upstream of phosphatidylinositol-3-kinase-Akt (PI3K-Akt) pathway were found in the wt-GISTs (Fig. 4B). Further analysis of the external dataset from Memorial Sloan Kettering Cancer Center (MSKCC) database including 137 GIST patients with 29 of them being wt-GIST confirmed that most frequently altered genes were NF1, EGFR, tumor protein p53(TP53), Succinate Dehydrogenase Complex Flavoprotein Subunit A(SDHA), Retinoblastomal 1(RB1), BRAF, Succinate Dehydrogenase Complex Flavoprotein Subunit B(SDHB), and TSC2, as shown in Fig. 4C. The analysis of KEGG pathways suggested that actin cytoskeleton, longevity regulating pathway and FoxO pathway indeed more frequently existed in the wt-GISTs. In addition, the Alzheimer disease (AD) pathway, Parkinson disease (PD) pathway, and insulin signaling pathway were present only in wt-GISTs in comparison to mu-GISTs (Fig. 4D).

## Discussion

In this study, we observed the incidence and severity of skeletal muscle atrophy being significantly higher in patients with wt-GIST when compared to the patients with mu-GIST. The sarcopenic nomogram constructed in our study showed the potential to be useful as a tool in predicting patients with wt-GIST before surgery. Our gene sequencing analysis of both our own data and the external data demonstrated that those aberrant gene signal pathways being closely associated with sarcopenia in the wt-GISTs more often than that in mu-GISTs. Our results indicated the potential application of CT-based sarcopenic assessment and gene analysis in pretreatment evaluation of GIST subtypes.

The prevalence of sarcopenia varied among different type of cancers^13^, and the incidence and clinical impact of sarcopenia on GIST are largely unknown. To date, the only published study on sarcopenia in GIST patients reported an incidence of 38.7% in a French cohort of GISTs (n = 31)^10^. However, our study showed a higher incidence of 56.32%. The reason for the discrepancy of incidence of sarcopenia between our study and their study^10^ was not clear. We speculate the difference in incidence might be due to different racial/ethnic background affecting body composition and muscle loss in patients with GISTs. Potential case selection bias should also be considered for both the French study and our study.

The sarcopenic nomogram constructed in our study, with the sarcopenic score (fitted by using age, gender and SMI) and with the MFI incorporated, showed potential value in differentiating wt-GIST from mu-GIST. The atrophy score represented the severity of a patient’s skeletal muscle atrophy after considering the effects of age and gender. The higher the score, the more severe the muscle atrophy. MFI represented the severity of septocutaneous adipose tissue. The higher the MFI, the more serious the adipose infiltration of skeletal muscle and the worse the muscle quality. Our higher incidence of sarcopenia and more severe muscle atrophy accompanied by adipose degeneration in patients with wt-GIST suggested that patients with wild-type tumors may have a significant decrease in muscle quantity and quality when compared to patient with mu-GIST^7,13^. Although sarcopenia could also occur in mu-GIST patients, our study showed that sarcopenia was an independent predictor for wt-GIST. In other word, sarcopenia could be a potential useful predictor for identifying wt-GIST before treatment.

The underlying genetic mechanism responsible for the different incidence and severity of sarcopenia between wt-GIST and mu-GIST is largely unknown. In search of the underlying molecule mechanism of sarcopenia in the wt-GIST, we analyzed the available gene data in our cohort, and extended this analysis to the external data in the Cancer Genome Atlas (TCGA). Regarding the most frequently observed genes in the wt-GIST, we identified five common genes abnormality including NF1, EGFR, BRAF, FAT1, IL-6ST and TSC2 in the wt-GIST. Patients with NF1gene deficiency have shown to have enhanced resting energy expenditure and respiratory quotient, indicating premature sarcopenia^14^. EGFR may be correlated with slow-twitch muscle phenotype and EGFR inhibitor may impede the loss of muscle slow-twitch fiber loss^15^. Fat1 is a protocadherin gene. It affects muscle patterning, regionalized muscle wasting and adult muscle fiber functions. The shape of muscle defects has been observed in adult fat1-knockout animal^16^. IL-6ST, also name gp130, is one of the common cytokine receptor subunits. It is shared by a series of IL-6 family which are well-known inflammatory cytokines, and function as prompting muscle wasting, stimulating protein catabolism and suppressing muscle synthesis^17^. Other studies have shown that TSC 2 phosphorylation activated by mTOR could be the response to the resistance training for the prevention and treatment of sarcopenia^18^. Collectively, these gene abnormalities involved in the total energy expenditure are correlated to food consumption and body composition, mitochondrial dysfunction, essential energy-producing processes, skeletal muscle function and cytokine-induced muscle catabolism, thus accelerating loss of muscle mass and function leading to sarcopenia^19^.

The causes of sarcopenia are likely multifactorial and molecular alterations may be present in complex signaling pathways^20^. Our results showed patients with wt-GIST had a higher incidence of mutant pathways associated with sarcopenia than patients with mu-GIST. For example, Longevity regulating pathways consisted of a series of genes and pathways for aging process have a high rate of sarcopenia^21–23^**.** FoxO proteins such as FOXO1 and FOXO3) are transcription factors which regulate the expressions of various atrophy-related genes atrogin-1 and MuRF-1 muscle, contributing to the decrease in muscle size and number^24,25^. Intriguingly, our study showed that the FoxO signal pathway was present in patients with mu-GIST in our own genetic data but this pathway was only present in the wt-GISTs in the external data. The exact reason for this discrepancy is not clear. We again speculate that the racial and ethnic differences between the cohorts may play a role.

Our study suggested that wt-GIST patients with sarcopenia had a shorter overall survival, as well as recurrence-free survival than wt-GIST patients without sarcopenia. Patients with recurrence all had sarcopenia. Our results were consistent with the literature indicating sarcopenia being a prognostic factor in cancer patients^26^. In advanced GIST, sarcopenia was correlated with shorter PFS and OS^27,28^. Further study with long-term follow-up is our on-going research.

There were limitations to this study. First, the retrospective nature of the study precluded the measurement of muscle strength which ought to be more important than muscle mass in terms of adverse health outcomes. Second, the absence of national cut-off points for muscle mass may hinder standardization of data and generalizability of results from sarcopenia research. Third, it was a single center study and the results may not be generalizable to other centers. Future study with a large-scale prospective multicenter approach is needed to validate our results and to expand this line of research.

## Conclusion

Our study identified higher incidence of sarcopenia, more severe muscle atrophy accompanied with more fat degeneration and more gene abnormalities in wt-GIST, than mu-GIST. Our study indicated that CT-based assessment of sarcopenia may be useful for differentiating wt-GIST from mu-GIST non-invasively before treatment. The molecule abnormalities underlying sarcopenia in wt-GIST may be potentially useful as alternative targets for detecting, mitigating and reversing sarcopenia.

## Supplementary Information

Supplementary Information.

## Data Availability

The original data will be made available to qualified the corresponding author of this paper upon request.

## Abbreviations

GIST: Gastrointestinal stromal tumor
mu-GIST: Mutant GIST
wt-GIST: Wild-type GISTs
CT: Computed tomography
PACS: Picture archiving and communication systems
VFA: Visceral fat area
MFA: Fat area in muscle
SFA: Subcutaneous fat
SMA: Skeletal muscle area
Hu: Hounsfield units
SMI: Skeletal muscle index
DFS: Disease free survival
PBL: Peripheral blood lymphocyte
NLR: Neutrophil lymphocyte ratio
NF1: Neurofibromatosis type 1
BRAF: B1 v-raf murine sarcoma viral oncogene homolog B1
KEGG: Kyoto Encyclopedia of Genes and Genomes
EGFR: Epidermal growth factor receptor
ARID1B: T-Rich Interaction Domain 1B
FAT1: FAT Atypical Cadherin 1
IL6ST: Interleukin 6 signal transducer
TSC2: Tuberous sclerosis 2
FoxO: Forkhead box O
PI3K-Akt: Phosphatidylinositol-3-kinase-Akt
MSKCC: Memorial Sloan Kettering Cancer Center
Tp53: Tumor protein p53
SDHA: Succinate Dehydrogenase Complex Flavoprotein Subunit A
RB1: Retinoblastomal 1
SDHB: Succinate Dehydrogenase Complex Flavoprotein Subunit B
AD: Alzheimer disease
PD: Parkinson disease
