# Supplementary material for: CT-based assessment of sarcopenia for differentiating wild-type from mutant-type gastrointestinal stromal tumor
Source: Sci Rep. 2023 Feb 24;13:3216. doi: 10.1038/s41598-022-27213-8 (PMC9958176; doi:10.1038/s41598-022-27213-8)
Supplement: Supplementary file 1 — Supplementary Information. [file 41598_2022_27213_MOESM1_ESM.docx]

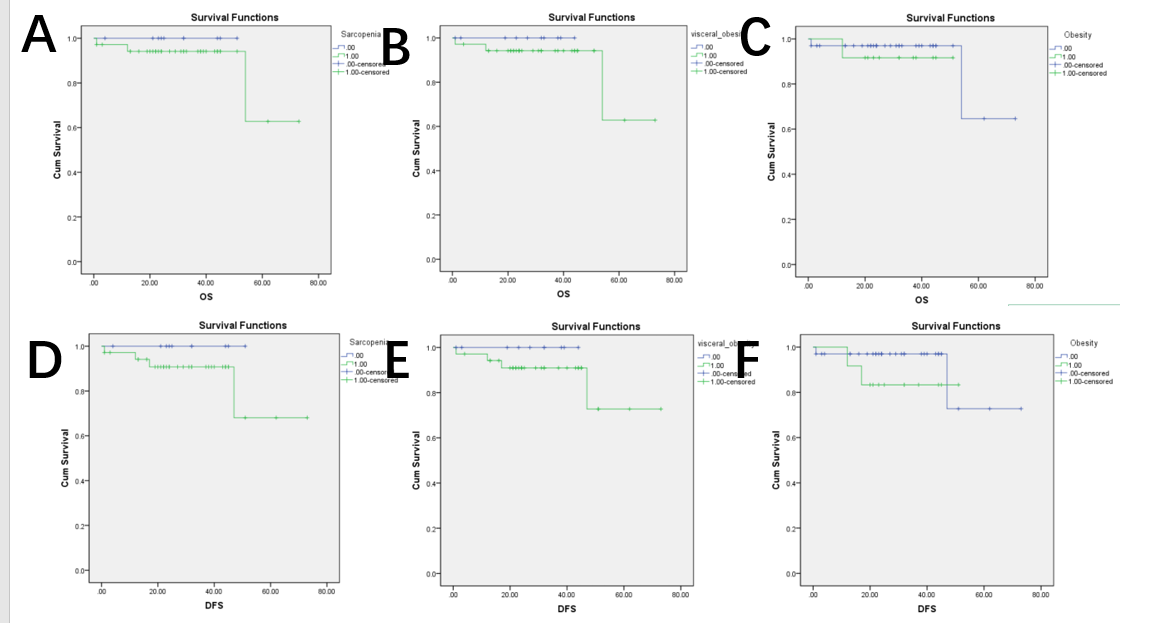


Supplementary Figure 1. Outcomes in patients with wt-GIST.

**Statistical analysis**

Statistical analysis was implemented with R software (http://www.Rproject.org). Multivariate binary logistic regression, nomograms, calibration plots, decision curve and correlation matrix plots were done with the “Caret”, “PROC”, “rmda”, “corrplot”, “forestplot”, “rms” and “corrplot” package. Decision curve was performed with the “rmda” package. And “survival” package was used for the survival analysis. The statistical significance levels were all two-sided with statistical significance set at 0.05. For the quantitative features, the Wilcoxon rank-sum test was used. For the qualitative features, Chi-square test or Fisher’s exact test were used to test differences between groups.

A logistic regression analysis was applied to identify independent predictors for wt-GIST and mu-GIST, and the individualized prediction model was developed by multivariable logistic analysis. The concordance index (c-index) and calibration curves were used to assess the performance of the nomogram. The diagnostic performance of the established models was quantified by the receiver operating characteristic curve and area under the curve(AUC). Decision curve analysis was conducted to validate the clinical usefulness of the nomogram by quantifying the net benefits at different threshold probabilities (Figure 2).

Analysis with univariate and multivariate cox proportional hazards was used to estimate Kaplan-Meier survival analyses and log rank test was used to explore the associations between features and clinical endpoints.

Numerical variables were summarized as the mean (standard deviation) and median (interquartile range). Categorical variables were reported as counts (percentage). An analysis of variance was used to compare continuous variables with symmetrical distributions across subgroups. Chi square tests and Fisher’s exact tests (n < 5) were used to compare categorical variables between subgroups. All tests of hypotheses were two-tailed and were conducted at a significance level of 0.05, and at a marginal significance level of 0.15. Statistical analyses were conducted using SAS 9.4.
